# Correlation of blunt cervical spinal cord injury magnetic resonance imaging tractography with the American Spinal Injury Association impairment scale motor scores

**DOI:** 10.4102/sajr.v25i1.2038

**Published:** 2021-04-01

**Authors:** Orapeleng Seboco, Fekade Gebremariam, Gina Joubert

**Affiliations:** 1 Department of Clinical Imaging Science, Faculty of Health Sciences, University of the Free State, Bloemfontein, South Africa; 2 Department of Biostatistics, Faculty of Health Sciences, University of the Free State, Bloemfontein, South Africa

**Keywords:** diffusion tensor imaging, cervical spine injury, spine trauma, ASIA, fractional anisotropy, subaxial cervical spine injury classification, apperant diffusion coefficient

## Abstract

**Background:**

The introduction of the Subaxial Cervical Spine Injury Classification system has created the need for a holistic imaging approach that encompasses both functional (neurological) and morphological information.

**Objectives:**

This study aimed to determine if there was a correlation between the blunt cervical spinal cord injury diffusion tensor imaging (DTI) fraction anisotropy (FA) value and the American Spine Injury Association (ASIA) impairment scale motor score.

**Method:**

Diffusion tensor imaging was performed on 26 patients with blunt cervical spine injury (all men with a median age of 46 years) admitted to the Pelonomi Tertiary Hospital spinal unit. Imaging was performed using the 1.5T Siemens Magnetom Aera machine’s built-in spine DTI protocol. Sagittal FA values were acquired at four different cervical spine regions (medulla oblongata, above the injury site, at the injury site and below the injury site).

**Results:**

Eight of the 26 patients had complete neurological fallout. Of the participants, 30% had injuries at the C4/C5 level, whilst injuries involving segments below and above C4/C5 affected 15% and 55% of participants, respectively. Injury site FA values (median 0.30) were significantly lower (*p* < 0.001) than the above injury site FA (median 0.46, *p* = 0.26) and below injury site FA (median 0.42 and *p* = 0.019). A significant correlation was noted between the injury site FA values and the ASIA impairment scale motor scores (*p* = 0.001, *r* = 0.87).

**Conclusion:**

FA value showed excellent correlation with the ASIA impairment scale motor scores.

## Introduction

Traumatic spinal cord injury can affect anyone, irrespective of age, gender or socio-economic background, with catastrophic social and economic implications. A South African study conducted in Cape Town by Philips et al.^1^ noted an incidence rate of 75.6 people per million spinal cord injuries in the public sector and 20 per million in the private sector, which is amongst the highest in the world, with the injuries mostly involving men (54%) between the ages of 18 and 30 years. The same study also noted that the cervical spine (C1–C8) was the most common site of spinal injury (53.1%), followed by the thoracic spine (38.6%) and lumbar spine (8.3%). Despite advances in diagnosis and treatment, traumatic spinal cord injury remains a huge problem, with 40% of traumatic injuries classified by the American Spinal Injury Association (ASIA A) (complete neurological fallout) and the remaining 60% having mixed neurological fallout (ASIA B, C and D). The lack of proper infrastructure in developing countries hinders proper social re-integration of the disabled.

The introduction of the ASIA in the mid-1970s helped facilitate the exchange of data and management ideas amongst healthcare providers involved in the management of traumatic spinal cord jury patients. The ASIA impairment scale is a standardised examination consisting of a motor examination, sensory examination and anal sphincter tone examination. The motor examination consists of grading five specific muscle groups in the upper extremities and five in the lower extremities, where each muscle is graded from zero (no power) to five (normal power). The sensory examination evaluates 28 specific dermatomes bilaterally for light touch (generally a piece of cotton) with a total score of 56.^2^

Magnetic resonance imaging (MRI) has long been considered an excellent complementary imaging tool to computed tomography (CT) in acute traumatic spine injury imaging because of its better soft-tissue resolution. The limitation of conventional MRI is the depiction of white matter as uniform tissue despite being composed of a complex array of directionally oriented nerve fibres, thus limiting its ability to detect finite axon pathology.^3^ *In vivo* methods to map the neurological pathways of the white matter have long been sought to increase the degree of pathology detection, with diffusion tensor tractography (DTT) imaging demonstrating excellent ability in depicting axon structural integrity. The highly directional architecture of the spinal cord has allowed diffusion tensor imaging (DTI) to accurately allow assessment of the spinal cord structural integrity by modelling the direction and magnitude of water diffusion.^3^ The ability of DTI to complement conventional spinal imaging by diagnosing subtle cord injuries, predict the need for early therapeutic intervention and monitor interventional outcomes has yet to be demonstrated.^4^

To our knowledge, only two human studies have reported DTI changes following acute cervical spine injuries,^4,5^ with the majority of studies conducted on animals.^6,7,8,9^ This study attempted to correlate apparent diffusion coefficient fractional anisotropy (FA) in a patient with acute blunt cervical spine injury with the ASIA impairment scale motor scores by using sagittal DTI imaging instead of the more commonly used axial views.

## Research method and design

### Design

A retrospective study was conducted on a cohort of 26 patients who were admitted to the Pelonomi Tertiary Hospital spinal unit in Bloemfontein, South Africa, following blunt cervical spine injury between 01 January 2018 and 01 December 2019. Patients with penetrating neck injury, younger than 18 years, patients scanned after 30 days post-injury and patients whose neurological examinations could not be assessed because of a low Glasgow Coma Scale were excluded from the study.

### Ethical considerations

Ethical approval to conduct the study was obtained from the University of the Free State Health Sciences Research Ethics Committee (HSREC) and permission to conduct the study at a state hospital was obtained from the Free State Department of Health (Ethics approval number: UFS-HSD2017/1299/2506). There was no consent needed in this study.

### Images protocol

All MR imaging was acquired with a 1.5T Siemens Magnetom Aera. Conventional MR imaging sequences included sagittal T2 (TE/TR, 33/5250 milliseconds [ms]), sagittal T1 (TE/TR, 9.5/1470 ms), sagittal PD (TE/TR, 9.9/3240 ms) and fluid-attenuated inversion recovery (TE/TR, 102/8000 ms). Diffusion tensor imaging images were obtained by using sagittal ep2d_diff_mddw (TE/TR, 82/2800 ms). A 12-channel head-neck array coil was used with a field of view from the base of the skull to the cervicothoracic junction.

### Data collection

**FIGURE 1:**
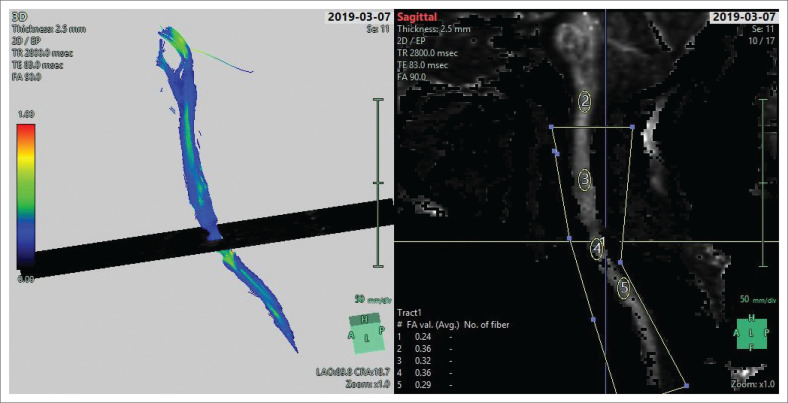
Sagittal cervical neck tractography with four levels of interest.

**FIGURE 2:**
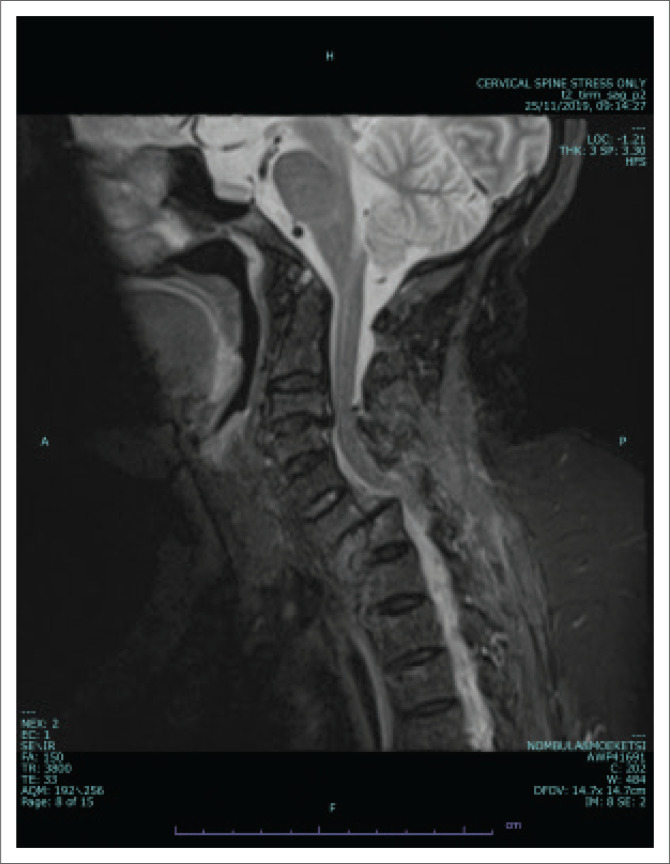
Cervical sagittal T2 weighted view.

Conventional sagittal MRI (T1/T2/PD with fat suppression) images were viewed for evidence of abnormal intramedullary spinal cord signal intensity to indicate the presence of a cord injury. Colour-coded DTI (Figure 1) was also evaluated for injury as depicted by alteration in spinal cord colour intensity. Fractional anisotropy values were obtained in the sagittal plane at four regions (the Medulla oblongata, the region above injury, the injury site and below the injury site) using an ellipsoid region of interest (ROI) with an area of 54 square millimetres (mm^2^). Figure 2 shows the sagittal T2 weighted image of a patient with acute blunt cervical spinal trauma.

Neurological assessment was performed by the spinal unit orthopaedics registrar using the ASIA impairment scale form and patients were classified as having complete or incomplete spinal cord injury.

### Statistical analysis

Numerical variables were summarised by means, standard deviations and percentiles; categorical variables by frequencies and percentages. Correlations were assessed using Spearman’s rank correlations. Independent numerical variables were compared using the Mann–Whitney test and paired data with the signed-rank test. Analyses were performed using SAS Version 9.4.

## Results

Diffusion tensor imaging tractography was performed on a total of 26 patients (all men with a median age of 46 years), with 30% of the patients having an injury at the C4/C5 level whilst injuries involving segments below and above C4/C5 affected 15% and 55% of patients, respectively. Following assessment with the ASIA impairment scale, eight of the participants were clinically classified as having complete neurological fallout, with the remaining 18 classified as having an incomplete spinal cord injury.

Of the 26 patients, one patient’s above injury site and medulla oblongata values were not obtained because of metal artefact from an upper cervical spine fixation and three patients had cervicothoracic junction injuries (DTI has poor resolution in the thoracic spinal cord), hindering evaluation of the below injury site FA readings (Table 1). Injury site FA values (median 0.30) were significantly lower (*p* < 0.001) than above injury site FA (median 0.46) and below injury site FA (median 0.42).

**TABLE 1:** Cervical region diffusion tensor imaging fractional anisotropy values.

Variables	Patient size	Standard deviation	10^−3^ mm^2^/s			
			Mean	Median	Minimum	Maximum
Medulla FA values	25	0.03	0.33	0.34	0.22	0.38
Above injury site FA	25	0.05	0.45	0.46	0.33	0.55
Injury site FA values	25	0.10	0.31	0.30	0.12	0.49
Below injury site FA values	23	0.60	0.40	0.42	0.23	0.49
Motor scores	26	36.40	68	85	10	100

FA, fractional anisotropy.

Injury site FA was compared to the medulla oblongata FA (*r* = 0.48, *p* = 0.016), below injury site FA (*r* = 0.58, *p* = 0.001) and above injury site FA (*r* = 0.47, *p* = 0.017) as noted in Table 2. The injury site FA values also had a significant correlation with the ASIA impairment scale motor score (*r* = 0.87, *p* = 0.001).

**TABLE 2:** Comparison of the findings using Spearman’s correlation coefficient (rs).

Variable	Medulla FA	FA above the injury site		FA injury site		FA below the injury site		Motor score	
		*r*-value	*p*-value	*r*-value	*p*-value	*r*-value	*p*-value	*r*-value	*p*-value
Medulla FA	1.00	0.25	0.21	0.48	0.01	0.1	0.43	0.28	0.15
FA above injury site	-	1.00	-	0.47	0.01	0.48	0.02	0.47	0.01
FA injury site	-	-	-	1.00	-	0.58	0.00	0.87	0.00
FA below injury site	-	-	-	-	-	1.00	-	0.49	0.01

FA, fractional anisotropy.

Patients with neurological fallout were divided into complete and incomplete neurological fallout based on their sacral motor and sensory findings (Table 3). Patients with complete spinal cord injury had an injury site median FA value of 0.18 (*p* < 0.001) and those with an incomplete spinal cord injury demonstrated a median value of 0.30 (*p* < 0.001).

**TABLE 3:** Breakdown of findings in patients with complete and incomplete neurological fallout.

Type of injury	Number of observations	Variables	Number	Median	Quartile	
					Lower	Upper
Complete	8	Medulla FA	7	0.34	0.31	0.35
		Above injury FA	7	0.41	0.41	0.46
		Injury site FA	8	0.18	0.15	0.25
		Below injury FA	5	0.33	0.28	0.39
		Time (in days)	-	7.50	2.50	11.00
Incomplete	18	Medulla oblongata FA	18	0.34	0.32	0.37
		Above injury FA	18	0.46	0.44	0.48
		Injury site FA	17	0.34	0.30	0.44
		Below injury site FA	17	0.42	0.41	0.44
		Time (in days)	-	5.00	4.00	10.00

FA, fractional anisotropy.

## Discussion

The Spine Trauma Study Group developed the Subaxial Cervical Spine Injury Classification (SLIC) after noticing a need for a more practical and comprehensive lower cervical spine classification system directly linked to clinical decision-making algorithms.^10^ The system is based on the evaluation of three major injury characteristics: (1) injury morphology, determined by the pattern of spinal column disruption on available imaging studies, (2) integrity of the discoligamentous soft tissue complex (DLC) represented by both the anterior and posterior ligamentous structures as well as the intervertebral disc and (3) the patient’s neurologic status determined by clinical assessment.^10^ Given the recent introduction of the SLIC score in managing patients with cervical spine trauma, the need for a holistic imaging protocol that can encompass both functional (neurology) and structural (anatomical) integrity has become crucial.

Imaging plays a critical role in traumatic spinal cord injury evaluation and management, with MRI universally acknowledged as an ideal non-invasive technique for examining acute spinal cord injury. Although conventional MRI plays a critical role in the diagnosis and management of spinal cord injury, it is neither sensitive nor specific in assessing spinal cord functional integrity. Chronic stenotic myelopathy (CSM) often appears normal on conventional MRI images even in patients with neurological fallout. Demir et al.^11^ noted that DTI (80%) had a high sensitivity when compared to T2 weighted images (60%) in detecting CSM. Diffusion tensor imaging indices (FA/ Apparent diffusion coefficient [ADC]) in CSM patients appear to depend on the degree of cord damage.^11^ It is apparent that DTI has a role to play in the pre-surgical planning for CSM patients, but the use of DTI in surgical intervention or in monitoring recovery is yet to be evaluated in detail.

Few studies have been conducted on healthy subjects to adequately establish a comprehensive database for cervical spine DTI parameters, thus hindering the clinical integration of DTI imaging. The studies that have been conducted on healthy subjects showed varying results, with Mamata et al.^12^ noting an average cervical spine FA value of 0.66 and Xiangshui et al.^13^ and Takashima et al.^14^ noting values of 0.74 and 0.64, respectively. Facon et al.^15^ noted that a precise measurement of FA values is difficult to obtain because they are affected by age, the measurement site, the imaging device and the imaging method used even in healthy spines. We attempted to overcome this inconsistency by comparing the injury site FA to normal adjacent cervical spine regions (region without DTI signal loss). Injury site FA values were demonstrated to be significantly lower than those obtained at the medulla oblongata, the level above and the level below the injury site FA. These indicated that changes in DTI FA values were a good marker of cervical cord injury.

Neural injury is characterised by demyelination, axonal and cell membrane disruption; this process leads to a reduction in FA and elevation of ADC values. Fraction anisotropy and ADC values in spinal cord injury have been correlated with several clinical assessment metrics, including the ASIA motor score. A study by Cheran et al.^5^ noted a good correlation between DTI fractional and ASIA impairment scale motor scores in patients with a non-haemorrhagic contusion on FA values measured on axial views. The objective of our study was to determine if it was possible to correlate DTI FA values with the ASIA impairment scale motor scores in patients with blunt cervical spine trauma using sagittal views. Similar to the study by Cheran et al.,^5^ our study also noted a good correlation between injury site FA values and the ASIA impairment scale motor scores (*p* = 0.001), with a marked difference in FA values between the patients with incomplete (0.30) and complete (0.18) neurological fallout.

No significant interval changes in the ADC values were noted in mild and moderate spinal cord injuries in a study conducted by Facon et al.,^15^ suggesting that ADC is not effective in assessing these injuries. The same study also showed a significant decrease in FA values 6 h post–trauma, whilst ADC values showed mild changes, suggesting that FA value is a sensitive marker for acute spinal cord injury and FA is more strongly related to the severity of the injury. Unlike in acute injury, chronic injury showed a significant increase in ADC values.

This study presented the following limitations that will need to be addressed with future studies and new policy implementations. Firstly, a large sample study is still needed to determine normal cervical spine DTI FA values. Secondly, the effects of time on the FA value still need to be addressed to determine the best imaging period. Thirdly, to facilitate large sample volume studies, a South African spinal trauma registry will need to be established. Li and Li^16^ found a good correlation between DTI image and histological variables at 72 h post-acute traumatic spinal cord injury, whilst our injury to scan time for the incomplete and complete spinal cord was 5.00 days and 7.50 days post–trauma, respectively, because of patient instability and logistical issues (difficulty in obtaining after-hours MRI).

## Conclusion

Diffusion tensor imaging is sensitive in detecting acute blunt cervical spinal cord injury, with FA values showing excellent correlation with the ASIA impairment scale motor scores. Given the importance of neurological severity in the management and prognosis of the cervical spine, this study highlights the need to include DTI in the conventional cervical spine trauma MRI imaging protocol.

This study should further contribute to the movement to incorporate and establish the DTI imaging technique as a crucial component of cervical spine trauma imaging.
